# Neurovascular unit protection—novel therapeutic targets and strategies

**DOI:** 10.1111/cns.13588

**Published:** 2021-01-13

**Authors:** Chen Chen, Peiying Li

**Affiliations:** ^1^ Department of Anesthesiology State Key Laboratory of Oncogenes and Related Genes Shanghai Cancer Institute Renji Hospital School of Medicine Shanghai Jiaotong University Shanghai China

Both acute brain injuries and chronic neurological disorders affect millions of patients worldwide every year, such as traumatic brain injuries, ischemic stroke, Alzheimer's disease (AD), and Parkinson's disease (PD). Due to the high mortality or catastrophic neurological and cognitive dysfunction of these brain injuries and neurological disorders, enormous efforts have been made to unravel the underlying pathophysiologic mechanisms in the past decades. Despite the respective progression in different cases, there are some notable commonalities among these different conditions, such as the cell‐to‐cell network.^1^ The concept of neurovascular unit (NVU) was proposed at the 2001 Stroke Progress Review Group meeting of the National Institute of Neurological Disorders and Stroke. ^2^ This concept sheds new lights on the cell‐to‐cell cross talk and the understanding of the crucial role of blood‐brain barrier (BBB) in the NVU.^3^ NVU is a functionally integrated cell group composed of vascular cells (endothelial cells, vascular smooth muscle cells, and pericytes), glia (astrocytes, microglia, and oligodendrocytes), neurons, and extracellular components. The components of NVU interact with each other while controlling BBB permeability and cerebral blood flow, as well as maintaining neuronal milieu.^4^ Dissecting the underlying mechanisms of the NVU dysfunction could pave the way for the exploration of novel therapeutic targets toward NVU dysfunction in multiple neurological diseases, especially with the rapidly evolving research techniques.

In this special issue, we collected nine research articles and four review articles, aiming to compile the recent research concerning the pivotal role of NVU and BBB in different neurological disorders. In three review articles, the topics mainly covered the mechanisms of neuroinflammation and BBB disruption in neurovascular diseases, especially in ischemic stroke. Wang LY et al.^5^ conducted a comprehensive review of the intimate connections between NVU and ischemic stroke. They not only summarized the roles of the NVU in the regulation of BBB integrity, cell preservation, inflammatory immune response, and neurovascular repair after stroke, but also mentioned the double‐sided role of the NVU in inflammatory immune responses to ischemic stroke. Besides the functional and phenotypic character of the NVU, they additionally included the influence of non‐coding RNAs in NVU on a transcriptional level. The comprehensive understanding of the NVU could pave the way for the exploration of therapeutic strategies targeting NVU dysfunction after stroke.

Huang XW et al.^6^ systemically reviewed the relationship between peripheral inflammation and BBB disruption. They emphasized the importance of protecting the integrity of BBB in maintaining the biochemical microenvironment of the brain. Meanwhile, the peripheral inflammation can induce BBB dysfunction, which could potentially contribute to the development of several central nervous system (CNS) diseases, such as neurodegenerative diseases AD, PD, multiple sclerosis (MS), and stroke. In addition, the authors also summarized the BBB disruption in COVID‐19–related CNS symptoms as well as in CAR‐T therapy–associated neurotoxicity.

Zhao FF et al.^7^ provided an inclusive review that endothelial glycocalyx (EG), the outermost layer of the blood vessel wall, acts as a physical and charge barrier and a mechanosensor, taking part in the regulation of vascular permeability, the modulation of inflammatory response, and anticoagulation. EG can be considered as the control center of BBB microenvironment. The structural disruption of EG can cause BBB dysfunction and neurological impairment, and thus, the mechanisms underlying the EG loss are attracting more and more attention. Restoring or maintaining the structure and function of EG represents an attractive therapeutic strategy for BBB protection and NVU functional preservation in neurological diseases.

In this special issue, we also assembled nine research articles, generally comprised topics over different neurovascular diseases, including both experimental and clinical research. Two research articles focus on ischemic stroke in animal models. Venkat et al.^8^ conducted their study with middle cerebral artery occlusion (MCAo) model in diabetic rats. They found that delayed treatment of vasculotide (VT), an angiopoietin‐1 mimetic peptide, could significantly improve the neurological function, promote vascular, and white matter remodeling and decrease inflammation in the ischemic brain after stroke in T1DM rats. In the study from Lei X et al.,^9^ they demonstrated that 2‐cyano‐3, 12‐dioxooleana‐1, 9‐dien‐28‐oic acid (CDDO)‐ethylamide (CDDO‐EA), a novel Nrf2 activator, could increase heme oxygenase‐1 (HO‐1) expression and attenuate cerebral ischemic injury by promoting microglia/macrophage polarization toward M2 phenotype using the same MCAo model in mice. Though the mechanisms of the neurovascular protection were different, they both used pharmacological strategies in neuroprotection against the ischemic injury.

Consistent with the review article by Wang LY et al, Akihiro et al.^10^ demonstrated the biphasic roles of pentraxin‐3 (PTX3) in cerebrovascular function after white matter stroke not only in mouse models, but also in postmortem human brain, suggesting that PTX3 could be a therapeutic target for white matter‐related diseases, including stroke. In addition, we also assembled another research article about the white matter injury (WMI) after traumatic brain injury (TBI). Mao LL et al.^11^ demonstrated that ethyl pyruvate (EP) could improve white matter remodeling in rats after TBI via modulating microglia polarization toward M2. The shift of microglia phenotype from pro‐inflammatory M1 to anti–pro‐inflammatory M2 could be a common strategy in different neurological diseases involving microglia and other components of NVU.

Intracerebral hemorrhage (ICH) could induce profound pathological changes in the NVU and leads to catastrophic consequences to patients’ neurological outcomes. It is important to identify the high‐risk patients. In a clinical research on spontaneous ICH, Zhu FP et al.^12^ utilized the big data and machine learning algorithms, the random forest to build models to predict coagulopathy in spontaneous ICH patients.

Collectively, this special issue gathers reviews and recent researches in the field of NVU protection under different pathological conditions. We anticipate that the novel findings in the current issue will give rise to a better understanding of the roles of NVU not only in the above‐mentioned neurological diseases, but also in those previously undervalued conditions, such as post‐operative cognitive decline or delirium. Meanwhile, future works are important to figure out the clinical relevance and translational perspectives in the mechanisms that underlying the NVU dysfunction after acute brain injury or neurological disorders, with a better use of the emerging high‐end and rapidly evolving technologies.

## References

[1] Zhang J , Badaut J , Tang J , Obenaus A , Hartman R , Pearce W . The vascular neural network–a new paradigm in stroke pathophysiology. Nat Rev Neurol. 2012;8(12):711‐716. 23070610 10.1038/nrneurol.2012.210 PMC3595043

[2] Iadecola C . The neurovascular unit coming of age: a journey through neurovascular coupling in health and disease. Neuron. 2017;96(1):17‐42. 28957666 10.1016/j.neuron.2017.07.030 PMC5657612

[3] Li Y , Zhu Z , Huang T , et al. The peripheral immune response after stroke‐A double edge sword for blood‐brain barrier integrity. CNS Neurosci Ther. 2018;24(12):1115‐1128. 30387323 10.1111/cns.13081 PMC6490160

[4] Zlokovic B . Neurovascular pathways to neurodegeneration in Alzheimer's disease and other disorders. Nat Rev Neurosci. 2011;12(12):723‐738. 22048062 10.1038/nrn3114 PMC4036520

[5] Wang L , Xiong X , Zhang L , et al. Neurovascular Unit: A critical role in the ischemic stroke. CNS Neurosci Ther. 2021;27(1):7‐16. 10.1111/cns.13561 PMC7804897 33389780

[6] Huang X , Hussain B , Chang J . Peripheral Inflammation and Blood‐Brain Barrier Disruption: Effects and Mechanisms. CNS Neurosci Ther. 2020;27(1):36‐47. 10.1111/cns.13569 PMC7804893 33381913

[7] Zhao F , Zhong L , Luo Y . Endothelial glycocalyx as an important factor in composition of blood‐brain barrier. CNS Neurosci Ther. 2021;27(1):26‐35. 10.1111/cns.13560 PMC7804892 33377610

[8] Venkat P , Ning R , Zacharek A , et al. Treatment with an Angiopoietin‐1 mimetic peptide promotes neurological recovery after stroke in diabetic rats. CNS Neurosci Ther. 2021;27(1):48‐59. 10.1111/cns.13541 PMC7804913 33346402

[9] Lei X , Li H , Li M , et al. The novel Nrf2 activator CDDO‐EA attenuates cerebral ischemic injury by promoting microglia/macrophage polarization toward M2 phenotype in mice. CNS Neurosci Ther. 2021;27(1):82‐91. 10.1111/cns.13496 PMC7804925 33280237

[10] Shindo A , Takase H , Hamanaka G , et al. Biphasic roles of pentraxin‐3 in cerebrovascular function after white matter stroke. CNS Neurosci Ther. 2021;27(1):60‐70. 10.1111/cns.13510 PMC7804900 33314664

[11] Mao L , Sun L , Sun J , et al. Ethyl Pyruvate improves white matter remodeling in rats after traumatic brain injury. CNS Neurosci Ther. 2021;27(1):113‐122. 10.1111/cns.13534 PMC7804862 33369165

[12] Zhu F , Pan Z , Tang Y , et al. Machine learning models predict coagulopathy in spontaneous intracerebral hemorrhage patients in ER. CNS Neurosci Ther. 2021;27(1):92‐100. 10.1111/cns.13509 PMC7804781 33249760

